# Integrating running water monitoring tools with the Micro Biological Survey (MBS) method to improve water quality assessment

**DOI:** 10.1371/journal.pone.0185156

**Published:** 2017-09-25

**Authors:** Lorenzo Traversetti, Francesca Losito, Alyexandra Arienzo, Ottavia Stalio, Giovanni Antonini, Massimiliano Scalici

**Affiliations:** 1 Department of Sciences, University Roma Tre, Rome, Italy; 2 INBB Interuniversity Consortium of Structural and Systems Biology, Rome, Italy; Universita degli Studi di Milano-Bicocca, ITALY

## Abstract

Running water habitats are among the most altered aquatic systems by human activities driving an increase in the organic components and the associated bacterial load as well. To contribute in improving the monitoring activities in running waters, here we tested the validity of the new Micro Biological Survey (MBS) method to specifically assess the bacterial load in running waters focusing on Total Viable Counts (at 22°C and 37°C) and *Escherichia coli* (at 44°C) in order to propose a new prognostic tool for watercourses. MBS method is an alternative colorimetric method for counting bacterial load in water and food samples that is easy to use and leads to a reliable and simple interpretation of results, being also faster and less expensive than traditional methods. Then, we compared MBS with the traditionally used reference method for the bacterial load, and with the most used biotic index for Italian watercourses based on the benthic invertebrates: the Extended Biotic Index (EBI). The last comparison was performed to validate the use of MBS in biomonitoring activities since the benthic invertebrate multi-species assemblage (and then EBI) alter own structure mainly depending on the organic component variation. During the first part of the study, the assessment of both linearity (regressions among bacterial concentrations) and accuracy (significant correlation between a measured value and a value used as reference) confirmed the validity of the MBS method. Second, the linear regressions between the three investigated microbial parameters vs. both physical-chemical descriptors and EBI, revealed the usefulness of MBS as a valid tool for routine microbiological analyses involved in rapid and easy field monitoring activities. This represents the first attempt to evaluate the river microbial status by exploiting the innovative MBS on running waters to propose it as new valuable monitoring tool in the biomonitoring field.

## Introduction

Inland waters are vital and vulnerable ecosystems that are critical for the sustenance of life globally. They are fundamental hydric resources for environmental, domestic, industrial and agricultural purposes [1]. However, inland waters are ecologically and economically deeply altered by human activities [2–4]. The Land-use and the urban development are two of the most relevant causes of the detrimental alteration of ecosystems’ structure, dynamic and functioning leading to effects on the health of biotic communities including humans [e.g. 5–8]. Although the wastewater treatment systems may decrease the organic contaminant concentration in watercourses, to date the great challenge is to prevent detrimental habitat exposures. For these reasons, the maintenance of healthy aquatic ecosystem throughout exploiting interdisciplinary approaches (the latter being widely stimulated by specific requirements of the Water Framework Directive–WFD 2000/60) represents a major concern in the environmental monitoring field [e.g. 9–13]. To date the latter is mainly based on the plant and animal biological diversity (by applying biotic indices) and physical-chemical properties. In this regard, a regular monitoring of water bodies is a critical tool to prevent the outbreak of diseases and occurrence of ecological hazards. It is also important for use-related purposes such as drinking water production, irrigation and recreation [1].

A progressive increase in the organic components and suspended materials in streams results in the heterotrophic bacterial load alteration [14]. The heterotrophic bacterial load may be interpreted as a good descriptor of organic pollution of surface waters, due to their rapid response to changes in environmental conditions [15]. Some of these heterotrophic bacteria grow in human and other warm-blooded animals’ intestine and faeces, and are known to be often pathogenic or potentially pathogenic. They are brought into aquatic environments through the release of wastewater effluents, surface runoff and soil leaching.

The detection and enumeration of all pathogenic microorganisms potentially present in the water is not practicable, because the isolation and identification of many of these is seldom quantitative and extremely complicated. Thus, nowadays, *Escherichia coli* is generally used to evaluate the degree of water sanitary quality, being a specific indicator of faecal pollution, since it occurs only the human and other warm-blooded animals’ digestive tract [16]. Despite the recognition of importance of microbial water quality for public health, only recently International Directives, like WFD and the New Bathing Water Directive (BWD; Directive 2006/7 of the European Commission, as evolution of the previous Directive 1976/160 of the European Economic Community) have emphasized the importance to evaluate the information on the microbial contamination.

The traditional microbiological culture-based methods are laborious, require dedicated laboratory space, expensive equipment and trained technicians. The Micro Biological Survey (MBS) method represent an attempt to propose a reliable alternative method that, as well as being accurate, reproducible and sensitive, is very easy to use, permits rapid assessment of water contamination and limits costs of instrumentation and personnel. This method surveys the catalytic activity of the redox enzymes in the main metabolic pathways of bacteria, allowing an unequivocal correlation between the observed enzymatic activity and the number of viable cells present in the samples [17]. The MBS analysis is performed using disposable and ready to use reaction vials, that contain the specific reagent for the analysis to perform. The test result is easy to interpret thanks to the verification of a colour change. The time required for colour change is inversely proportional to the bacterial charge present in the sample: the presence of a high concentration of bacteria could be seen in few hours in comparisons to the 24–48 hours needed by traditional methods. MBS method has been already demonstrated that this method is a valid and accurate tool to evaluate microbiological quality of food, and biological and environmental matrices [17–20].

Since MBS was just used for drinking waters [20], the present study extends its use to the evaluation of microbial loads in other type of matrices, such as running waters, specifically in central Italy. In this case, it was necessary to make a new specific calibration for freshwater, in order to test the MBS method as a novel tool for the bio-assessment of the environmental health associated to human activities. Indeed, such integrated approaches to provide direct information on the ecological status of freshwaters as well as the potential risks for humans’ health are little exploited, although their use is openly encouraged.

To date indirect information on the organic load into running waters may be extrapolated by using traditional biotic indices. Such indices describe the effects of the increasing organics as a variation of the structure of selected invertebrate multi-species assemblages. Anyway biotic indices are not able to provide a direct evaluation on the bacterial load. Then, MBS outputs may fill this gap. Indeed in this study we test some overlap with the widely applied biotic index for Italian freshwaters: the Extended Biotic Index (EBI, accordingly modified for Italian running waters by [21]). This may emphasize the usefulness in exploiting both bacteria and riverine invertebrates for obtaining a wider definition of the running water environmental status [22]. Additionally, comparing MBS *vs*. EBI allows to propose a new early warning system in the light of the WFD requirements and according to the guidelines for HORIZON 2020. The early warning systems are rapid, effective-cost signal alarms useful for monitoring activities that have necessarily to be integrated with analytical methods to evaluate the potential environmental damages, when risks are identified. Nowadays, although in Italy in the last years a new multimetric index has been proposed to assess the ecological status of river ecosystems, EBI represents a good descriptor of the organic pollution in freshwaters [21–24].

In this context, our study aimed to test the validity of the MBS method in assessing the running water quality (i.e., the microbial load) to (1) propose it as an early warning system of the organic pollution of human source in riverine habitats, and (2) use it as a new innovative monitoring tool in the biomonitoring field for freshwaters. It is also an essential tool to ascertain hygienic quality of water sources for human consumption and for general community purposes. To do so, MBS has been compared with EBI.

## Material and method

### Study area and environmental surveys

**Fig 1:**
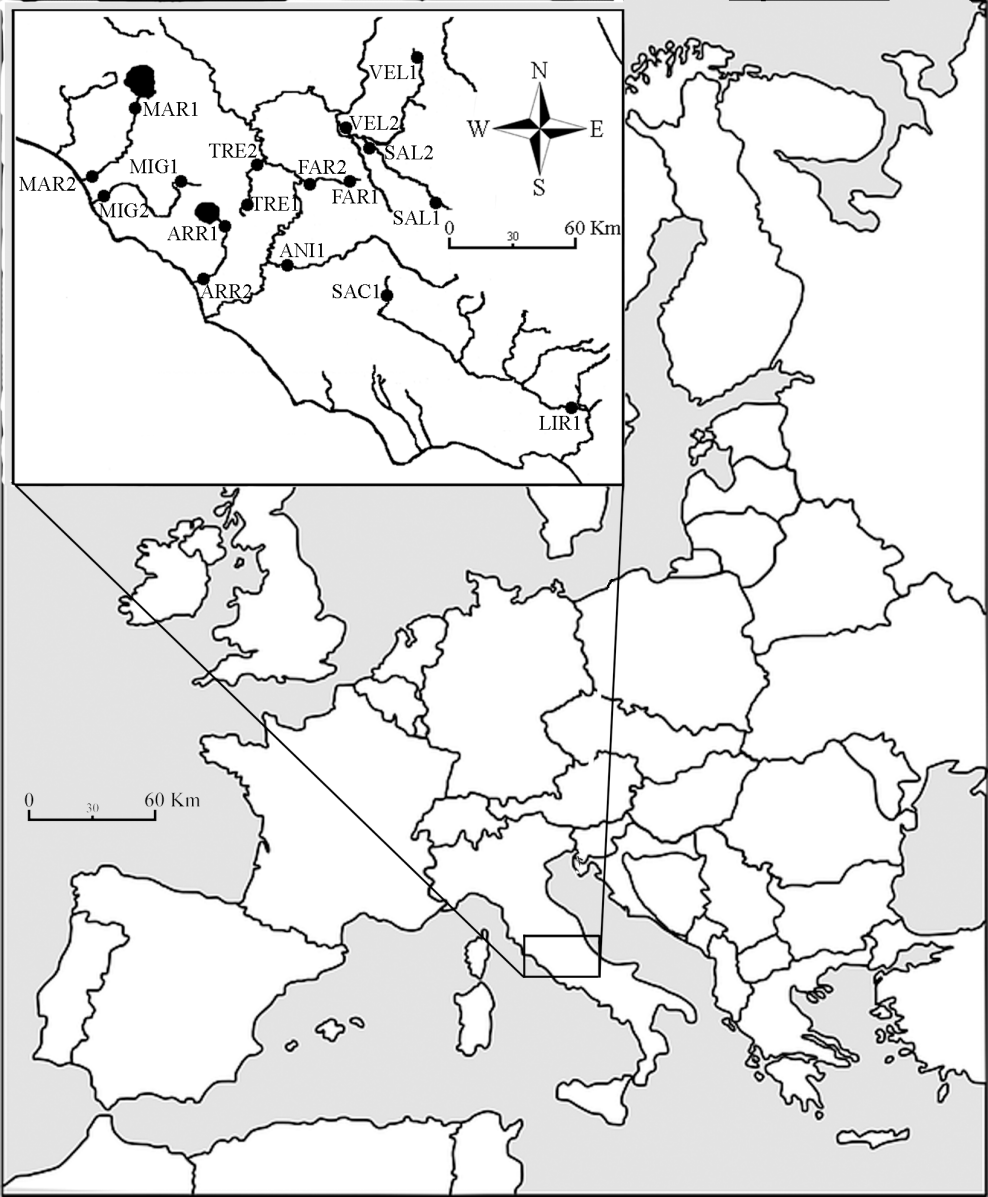
Location of the sampling sites within the study area. Marks: ANI1 = site on the River Aniene; ARR1 = first site on the River Arrone; ARR2 = second site on the River Arrone; FAR1 = first site on the River Farfa; FAR2 = second site on the River Farfa; LIR1 = site on the River Liri; MAR1 = first site on the River Marta; MAR2 = second site on the River Marta; MIG1 = first site on the River Mignone; MIG2 = second site on the River Mignone; SAC1 = site on the River Sacco; SAL1 = first site on the River Salto; SAL2 = second site on the River Salto; TRE1 = first site on the River Treja; TRE2 = second site on the River Treja; VEL1 = first site on the River Velino; VEL2 = second site on the River Velino.

We investigated 17 sites located in 10 different streams of Latium, central Italy, one of the most hydro-morphologically heterogeneous region [25] (Fig 1, Table 1). Before collecting waters and sampling, two physical-chemical descriptors were evaluated in all sites by the multi-parameter field probe WTW Multi340i (WTW, Weilheim): conductivity (C, μS/cm), temperature (T, °C). 500 ml of water samples were stored at 4°C into a portable refrigerator Gio’Style Shiver 26 (Gio’Style, Urgnano: IT) and rapidly transferred to the lab, and analysed for chemical oxygen demand (COD, mg/l), nitrates (NO3-, mg/l), total phosphorus (P, mg/l) by using the spectrophotometer WTW MPM 3000. In addition, water velocity (VEL, cm/s) was recorded for each site with a mechanical flowmeter General Oceanics 2030 (General Oceanics, Miami; FL).

**Table 1:** Geographic coordinates and name of locations and sampled rivers. The use of numbers 1 or 2 in marks depend on the part of river that was sampled: 1 –sampling site into the first 5 km from the source; 2—sampling site into the last 5 km till the mouth or access into another water course. The only exception is represented by the River Aniene where site ANI1 is located into the last 5 km till the access into the River Tiber.

	River name	Location name	Latitude	Longitude
ANI1	Aniene	Rome	41°55'36.79"N	12°40'6.65"E
ARR1	Arrone	Osteria Nuova	42° 2'7.35"N	12°18'39.21"E
ARR2	Arrone	Fregene	41°52'10.51"N	12°10'58.91"E
FAR1	Farfa	S. Mary Mountain	42°13'53.17"N	12°46'6.61"E
FAR2	Farfa	Ponte Sfondato	42°12'28.60"N	12°38'11.40"E
LIR1	Liri	Balsorano	41°47'31.28"N	13°34'28.40"E
MAR1	Marta	Marta	42°31'1.23"N	11°55'8.92"E
MAR2	Marta	Tarquinia	42°15'45.12"N	11°45'28.53"E
MIG1	Mignone	Vejano	42°12'51.27"N	12°6'43.56"E
MIG2	Mignone	S. Mary house	42°14'26.25"N	11°54'40.18"E
SAC1	Sacco	Gimignano	41°49'47.19"N	13° 0'40.25"E
SAL1	Salto	Civitella	42°12'34.13"N	13°11'1.68"E
SAL2	Salto	Rieti	42°21'45.83"N	12°55'34.78"E
TRE1	Treia	Gelato Mountain	42°11'1.96"N	12°22'39.89"E
TRE2	Treia	Civita Castellana	42°16'55.16"N	12°25'28.57"E
VEL1	Velino	Posta	42°31'19.47"N	13° 7'10.05"E
VEL2	Velino	Rieti	42°23'39.85"N	12°52'49.25"E

### Microbiological parameters

#### MBS method procedure

The MBS analytical procedure starts from the dissolution of the MBS reagent with 10 ml of sterile distilled water [17]. Once the vials are hydrated, the protocol requires the inoculation of the sample (1 ml), and then the incubation at the given temperature, depending on the kind of analysis to perform. It can be used also a thermostatic optical device that maintains the given temperature of incubation (37° or 44°C). In this study, the analyses were carried out using TVC and *E*. *coli* vials for the detection and quantification respectively of heterotrophic bacteria and *E*. *coli*.

Since results of the heterotrophic tests are usually expressed as the number of colony forming units per milliliter (CFU/ml), 1 ml of water from each site was directly inoculated in the MBS TVC vials. After inoculation, the TVC vials for the detection of heterotrophic bacteria that may be derived from environmental sources were incubated at 22°C in a thermostat. The TVC vials for the detection of heterotrophic bacteria that may be derived from human or animal sources were instead incubated at 37°C in the optical reader. A positive result (that means the presence of bacteria load in the sample) corresponds to a color change from blue to yellow within for 48 hours for the TVC vials at 22°C and 24 hours for the TVC vials at 37°C.

For *E*. *coli*, outputs were evaluated and expressed as the number of colony forming units for 100 ml (CFU/100 ml), as indicated by the law DL 152/2006. For this reason, the MBS analyses of *E*. *coli* were carried out on 100 ml using a filtering device made of a polycarbonate body provided with a polycarbonate filter of 0.45 μm, as described in [20]. After the filtration of 100 ml of water sample, the filter was inserted into the MBS COLI vials and incubated at 44°C in an optical reader that automatically detects the change of color of the vials. A positive result corresponds to a colour change from red to yellow within 48 hours.

The reliability of the method was tested and demonstrated in a previous study, in which an R&R gage study was conducted to compare different methods confirming that a laborious analytical protocol, a significant sample pretreatment together with an individual interpretation of results, more probably lead to human error [26].

### Reference method

The analyses of water samples with the reference method for the detection and quantification of heterotrophic bacteria were carried out using the poured plate technique on Water Plate Count Agar (Oxoid, Rodano, Italy). The inoculated plates were incubated for 22±2°C for 68±4h and at 37±2°C for 44±4h according to ISO 6222:1999.

The analyses of water samples with the reference method for the detection and quantification of *E*. *coli* were carried out using the Most Probable Number (MPN) technique. This technique is composed by three tubes containing Lactose Broth (Liofilchem, Roseto degli Abbruzzi, Italy) in which were inoculated for each dilution 10 ml, 1 ml, and 0,1 ml of the samples and incubated at 35°C±0.5°C for 48±3h. From each gassing lactose broth tube, a loopful of the suspension was transferred to a tube of E.C.+MUG Broth (Liofilchem, Roseto degli Abbruzzi, Italy) and incubated at 44.5°C±0.2°C for 24±2h. The presence of growth (turbidity) and a bright blue fluorescence under a long-wave (366 nm) UV light (with or without the production of gas) were considered confirmatory for the presence of *E*. *coli*. The most probable number was calculated using the specific MPN table (ISO 9308–2:2012).

### Benthic invertebrate collection

In each site, the sample collection was carried out by kicking the riverbed along a linear transect from the two banks and collecting specimens with a standard net (25 × 25 mm frame, mesh 500 μm). Benthic invertebrates were grossly sorted in field, preserved with 80% ethanol and then identified in laboratory to family level (only Ephemeroptera, Irudinea, Plecoptera, Odonata and Triclada were identified at genus level) based on [27] taxonomic guide. All families (or genus when identified) will be called Sistematic Units (SU) hereafter. EBI was calculated based on the benthic invertebrate SU. Particularly the index values (varying from 14 for oligotrophic waters to 1 for euthropic) can be obtained as the number located at the intersection between row and column by using a double entry matrix with the SU on the y axes (top down less sensitivity to environmental alteration) and the total number of SU x site on the x axes (S1 Fig) [21]. This value can be converted into five quality class judgements: very good (I; from 14 to 10); good (II, from 9 to 8); sufficient (III, from 7 to 6); bad (IV, from 5 to 4); very bad (V, from 3 to 1).

### Data elaboration and statistical analysis

#### Calibration of the MBS method

Water samples coming from different streams of Latium were analysed both with the MBS method and the reference method. The results were than compared to determine the linearity and accuracy of the MBS method for the determination of the parameters heterotrophic bacterial count at 22°C, heterotrophic bacterial count at 37°C, and *E*. *coli* at 44°C in inland water samples [20]. The linearity was evaluated by plotting bacterial concentrations obtained with the reference method (expressed as the log of CFU/ml o CFU/100ml) with the time for taken place colour change of the identical water samples analysed with the MBS method (expressed as hours) for all the parameters. Then, all these concentrations were plotted with the corresponding ones obtained with the reference method to demonstrate the accuracy of the MBS method.

#### Environmental parameters and Extended Biotic Index *vs*. MBS

In order to find out the relationship between environmental and microbiological parameters, a series of Spearman correlations were carried out between the selected environmental descriptors vs. (1) heterotrophic bacterial count (both 22°C and 37°C) and (2) *E*. *coli* (44°C).

Finally, in order to estimate the relationship among EBI and the three microbial parameters, a series of linear regressions were performed, specifically: a) EBI vs. heterotrophic bacteria count at 22°C; b) EBI vs. heterotrophic bacteria count at 37°C; c) EBI *vs*. *E*. *coli* at 44°C.

All statistical analyses were performed with Statistica 8 Stat. Soft. and PAST package ver. 1.94b. In all cases, the level of confidence (α) was always set at 0.05.

## Results

### Linearity and accuracy of the MBS method for the determination of the parameters heterotrophic bacteria count at 22°C and 37°C and *E*. *coli* in inland water samples

The analysis of water samples directly collected from rivers was performed as previously described using both the MBS method and the reference method for the determination of the parameters heterotrophic bacteria count at 22°C and 37°C and *E*. *coli*. In order to demonstrate the validity of the results obtained with the MBS method to use to find out the relationship between environmental and microbiological parameters, linearity and accuracy for this method were assessed.

**Fig 2:**
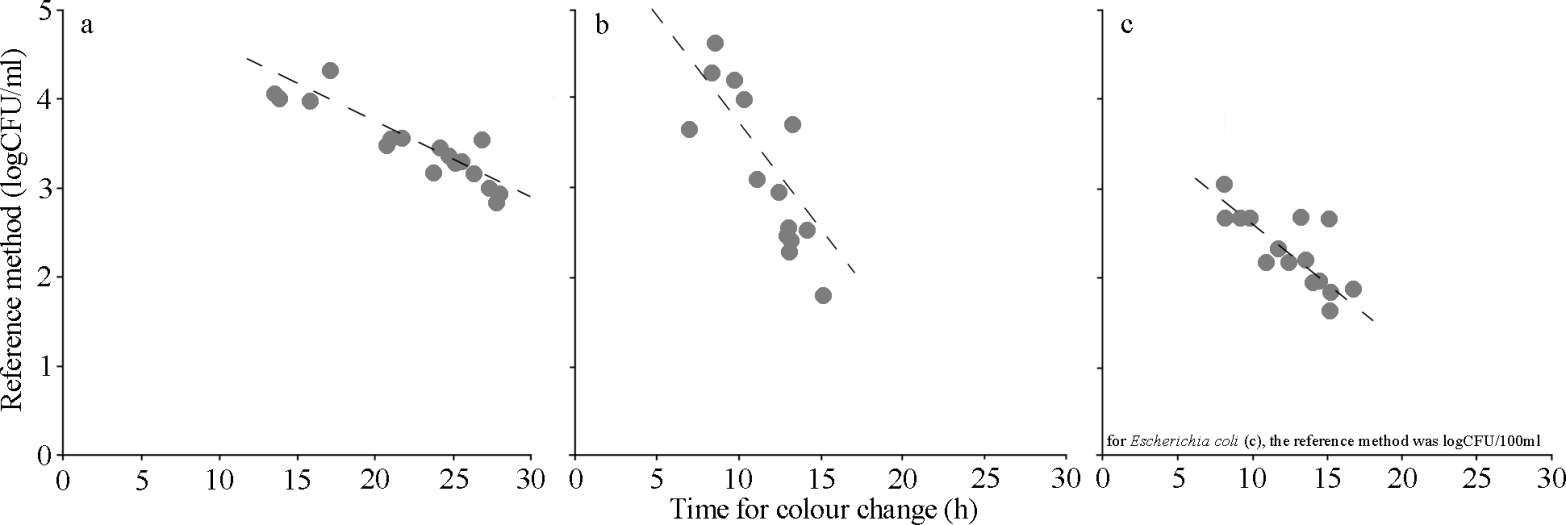
Linearity. Correlations between bacterial concentrations (expressed ad logCFU/ml, obtained with the reference method) and the time occurred for color change concentrations (expressed as hours, h, obtained analyzing identical surface water samples). Continuous line linear regression analysis: R^2^ = 0.81 heterotrophic bacteria count 22°C, R^2^ = 0.79 for heterotrophic bacteria count 37°C, R^2^ = 0.87 for E. coli. Each point is the mean of at least three different analyses. a = heterotrophic bacteria count 22°C; b = heterotrophic bacteria count 37°C; c = *E*. *coli*.

Linearity is the ability of the method when used with a given matrix to give results that are in proportion to the amount of analyte present in the sample, that is, an increase in analyte corresponds to a linear or proportional increase in results as indicated by ISO16140 (2003). The trend line equations and their correlation factors were calculated (R^2^ = 0.81 heterotrophic bacteria count 22°C, R^2^ = 0.79 for heterotrophic bacteria count 37°C, R^2^ = 0.87 for E. coli, P <0.001) (Fig 2, S1 Table).

**Fig 3:**
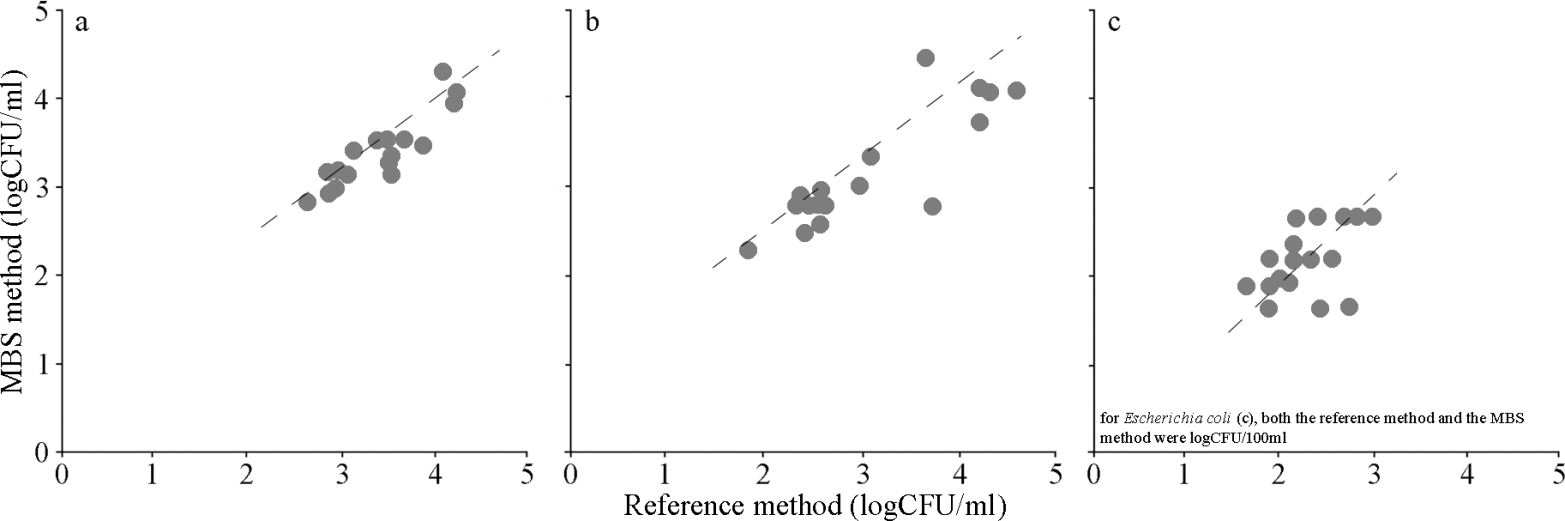
Accuracy. Correlations between bacterial concentrations (obtained with the reference method) and the MBS method (analyzing identical surface water samples), both expressed as logCFU/ml. The continuous lines represent the theoretical perfect correspondence between the two analysis. Continuous line linear regression analysis: R^2^ = 0.80 heterotrophic bacteria count 22°C, R^2^ = 0.74 for heterotrophic bacteria count 37°C, R^2^ = 0.72 for *E*. *coli*. Each point is the mean of at least three different analyses. a = heterotrophic bacteria count 22°C; b = heterotrophic bacteria count 37°C; c = *E*. *coli*.

The trend line equations were used to calculate, from the times for color change of MBS, the bacterial concentrations (expressed as the log of CFU/ml o CFU/100ml). Accuracy is the degree of correspondence between the response obtained by the reference method and the response obtained by the alternative method on identical sample (ISO 16140). The straight lines obtained were close to the ideal y = x (slope = 1.00), with values of correlation factor which further confirm the high equivalence between the reference method and the alternative MBS method (R^2^ = 0.80 heterotrophic bacteria count 22°C, R^2^ = 0.74 for heterotrophic bacteria count 37°C, R^2^ = 0.72 for *E*.*coli*, p<0.01). For this reason, MBS bacterial concentrations were used hereafter (Fig 3, S1 Table).

### Environmental parameters and Extended Biotic Index *vs*. MBS

After the collinearity test, the following 5 environmental descriptors were selected: C, CON, P, T, VEL (see S2 Table for details in physical-chemical descriptors). The R values for the Spearman correlations between microbiological parameters and environmental descriptors are shown in Table 2. It is possible to observe a significant correlation between the three microbiological parameters and the three physical-chemical descriptors C, T, and VEL. Particularly the used microbiological parameters were always positively related with C and T, and negatively with VEL.

**Table 2:** Correlation coefficients values (R2) between all the selected physical-chemical descriptors and Total Viable Count (that is heterotrophic bacteria count) at 22 and 37°C, and *E*. *coli* at 44°C (significant correlations at p<0.05 are in bold). Marks: COD = chemical oxygen demand (mg/l); C = conductivity (μS/cm); P = total phosphorus (mg/l); T = temperature (°C); VEL = velocity (cm/s).

	heterotrophic bacteria count 22°C	heterotrophic bacteria count 37°C	*E*. *coli* 44°C
**COD**	0.21	0.10	0.17
**C**	**0.44**	**0.47**	**0.53**
**P**	0.31	-0.16	0.09
**T**	**0.53**	**0.44**	**0.60**
**VEL**	**-0.56**	**-0.60**	**-0.68**

EBI revealed a general altered status of sampled sites mainly due to anthropogenic pressures insisting on rivers. In particular, no studied sites were included within the first water quality classes (very good) and the major part of them ranged from the water quality class III (sufficient) to IV (bad) (S3 Table).

**Fig 4:**
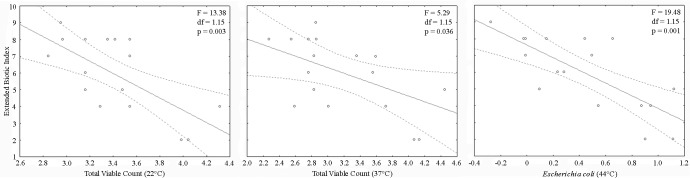
Linear regressions between the Extended Biotic Index (EBI) and the three analysed microbiological parameters, expressed as Total Viable Count (TVC) at 22°, 37°, and 44° C (the latter for *E*. *coli*). Dotted lines correspond to the 95% confident intervals.

As for the regressions between all the microbiological parameters and the EBI values, significant negative relationships were always obtained highlighting the closeness of MBS and EBI (Fig 4).

## Discussion

The main aim of this study was to demonstrate the effectiveness of the alternative colorimetric MBS method to evaluate the microbiological status of running waters. Moreover, the easy analytical procedure of this method does not require the presence of an equipped laboratory nor of skilled technicians, so the analyses can be carried out directly on field or in close proximity of the surface water to be analysed. Indeed, the MBS method has several advantages over traditional method for microbiological analysis. Above all, it is faster, cheaper and the possibility of being used by everyone (without specific skills) and everywhere (without a laboratory) leading to the possibility of being used in close proximity of the surface water to be analysed. The presence of fecal contamination in running water and the identification of the contamination source is important in order to assess the risk of exposure to human health. This allows to better preserve inland water resources by planning preventive measures and assessing bioremediation actions [28]. The quantitative MBS method for heterotrophic bacteria count at 22°C and 37°C and *E*. *coli* showed high linearity and accuracy. According to [17], the high linearity was confirmed by the significant linear inverse relationship between the MBS times occurred for the color change of the vials and the bacterial concentrations by the reference method [24]. In addition, the linear regression between MBS bacterial concentrations *vs*. reference method bacterial concentrations confirmed a significant accuracy between the two selected microbiological methods. As a consequence, the validity of the MBS method as an alternative and fast microbiological method can be highlighted, as just tested in previous studies for food [17, 24] and biological matrices [18].

The evaluation of physical-chemical parameters confirmed previous studies that showed a relevant influence to a series of factors including inputs from exogenous sources and carbon and nutrient availability on bacteria in river systems [29–31]. The evaluation of the organic load into waters can be extrapolated by using biotic indices, which describe the effects of the increasing organics as a variation of the structure of selected biological assemblages. Then, the last step of this study was oriented to overlap MBS outputs to EBI. [23] showed the existence of a direct impact of organic enrichment on the benthic invertebrate communities of a river through a study confronting sites which were situated upstream and downstream of several sewage treatment works [32]. Besides this, [19] showed the existence of a significant correlation between *E*. *coli* and EBI, this allowing to emphasize the importance of using both information to obtain a wider definition of the environmental status of a river [22].

Through the regressions MBS *vs*. EBI, the colorimetric method functionality in assessing the river degree of organic pollution was tested. The significant negative regression of all microbiological with EBI obtained with our findings confirmed the strict link existing between these descriptors. This led to the proposal of this method as a rapid and valuable indicator of the human exploitation by the domestic wastewater release, confirming the key role of both the microbial descriptors and the EBI to evaluate the organic load as an indirect way of testing the human organic pressures insisting on rivers. Our findings suggest that the MBS method could be a valid tool for routine microbiological analyses involved in a more rapid and easy use in field monitoring activities.

Although several data suggest that a limitation of this method applied to food and water analyses can arise (since MBS requires verification and, in some cases, the generation of a new calibration curve specific for the analysed matrix), our findings confirm previous results in both rivers and costal watershed ecosystems, which revealed a direct influence of several human alterative factors, such as wastewaters or sewage treatment plants on the microbiological and organic concentrations into inland waters [15, 32]. This is probably related to different stressor sources (such as dams, water intakes, wastewaters) insisting on central Italy rivers due to the river human exploitation [8, 25]. Particularly, the effect of wastewaters [20, 33–35] and sewage treatment plants [36–38] in altering the organic load into rivers have been studied as well. [38] showed how the release of sewage treatment plants causes an increase of faecal indicator bacteria concentrations, more pronounced for *E*. *coli*, that reaches the highest level during the first 30 minutes of discharge.

## Conclusions

This study represents the first attempt to evaluate the river microbial status by exploiting the innovative Micro Biological Survey (MBS) method on water samples of central Italy, with the aim to provide easily and quickly more detailed information about water health conditions. MBS results compared well with both the traditionally used reference method for the bacterial load and with the most used biotic index for Italian watercourses based on the benthic invertebrates, leading to the conclusion that use of MBS method can simplify future monitoring activities to test the microorganisms load into inland waters, allowing more expeditious and useful monitoring strategies for river management.

## Supporting information

S1 Fig Reference double entry matrix to calculate the Extended Biotic Index (EBI) value and the correlated water quality class. (TIF)

S1 Table Linearity (above) and accuracy (below) for the detection of heterotrophic bacteria count 22°C, heterotrophic bacteria count 37°C and *E*. *coli*. Above: bacterial concentrations (expressed as log CFU/ml) obtained with the reference method and corresponding times (expressed in hours) occurred for colour change obtained with the MBS method analyzing identical surface water samples. Below: bacterial concentrations (expressed as log CFU/ml) obtained with the reference method and corresponding bacterial concentrations (expressed as log CFU/ml) obtained with the MBS method analyzing identical surface water samples. Each value is the mean of at least three different analyses.
(DOC)

S2 Table Values of the environmental parameters collected within the study area per investigated site. Marks: BOD = biochemical oxygen demand (mg/l); C = conductivity (μS/cm); COD = chemical oxygen demand (mg/l); NH_4_^+^ = ammonium (mg/l); NO_3_^-^ = nitrates (mg/l); P = total phosphorus (mg/l); SAT = saturation (%); T = temperature (°C); VEL = velocity (cm/s). For site marks, see the Fig 1 caption.
(DOC)

S3 Table Complete list of macroinvertebrates collected within the study area. Protocols followed national standards and no protected taxa were collected.
(DOC)
