# Expression of Concern: Integrating running water monitoring tools with the Micro Biological Survey (MBS) method to improve water quality assessment

**DOI:** 10.1371/journal.pone.0328284

**Published:** 2025-07-16

**Authors:** The PLOS One Editors

After this article [1] was published, concerns were raised regarding undisclosed potential competing interests. Based on information gathered in editorial follow-up, the updated competing interests statement is: GA is inventor of the Micro Biological Survey (MBS) method, a colorimetric method for counting bacterial load. This article [1] evaluates the validity of the MBS method for assessing bacterial load in running water. GA is listed as an inventor of patent EP2041297 (for a colorimetric bacterial load detection method); MBS SRL, a spin-off company of Roma Tre University that commercializes products based on MBS method, is the current assignee and owned the patent at the time of publication of [1]. GA supervises technical activities of MBS SRL on behalf of Roma Tre University, but does not receive financial support from MBSSRL. At the time of publication of this article [1], GA was a shareholder of MBS Diagnostics Limited, which developed an MBS-based test for use in urinary tract infections; MBS Diagnostics Limited is the assignee of patent EP3433606A1.

GA disagrees that the above information falls within the scope of the interests that must be declared for this article per the journal’s editorial policy.

During editorial follow-up, a member of the *PLOS One* Editorial Board raised the following concerns with regards to the methodology and limitations in [1] which were not resolved upon discussion with the authors:

- The study does not appear to account for potential variability in different water matrices.
- The study uses linear regression to establish the validity of MBS but does not provide Bland-Altman plots or range-specific error analysis, and as such the study does not appear to show how MBS performs at different bacterial concentration ranges.
- Normality tests do not appear to have been performed before choosing Spearman’s correlation.
- On page 7 (Environmental parameters and Extended Biotic Index vs. MBS), it is stated that “The R values for the Spearman correlations…,” however, R applies to Pearson’s bivariate correlation, and the correlation coefficient is denoted by ρ for Spearman’s.
- The negative correlation between MBS and EBI could reflect independent responses to pollution rather than true equivalence.

The last author provided the following clarifications around the limitations of [1]:

- The analyses were conducted with the environmental descriptors indicated because, in previous exploratory analyses, other descriptors usually detected during environmental monitoring campaigns (such as dissolved oxygen, pH, and turbidity) were found to be highly correlated with each other.
- More measurements in the same conditions would ideally have been repeated to understand the margin of error, but the constant unidirectional flow (whose flow rate and speed being dependent on precipitation, catchments and saturation of the aquifer) which, carrying everything downstream, constantly changes the local conditions meant this was not possible. This limitation was overcome by collecting more data in more conditions.
- The main objective of [1] was to propose an initial possibility of verifying whether MBS could be in line with what was described by macroinvertebrates in the case of evaluating the ecological quality of running waters. The term “early” refers to proposed systems that can identify rapid responses from the biotic component, and whether, following the arrival of a disturbance, there is a component of the biota that can turn on the alarm before other components, once the disturbance has already arrived. Early refers to the response speed of the biomodel used once the disturbance has already started rather than being predictive.

The *PLOS One* Editors issue this Expression of Concern to make readers aware of the limitations of [1] and the unresolved methodological concerns listed above, as well as the information about undeclared potential competing interests.
