# Supplementary figures and images for: Integrating running water monitoring tools with the Micro Biological Survey (MBS) method to improve water quality assessment
Source: PLoS One. 2017 Sep 25;12(9):e0185156. doi: 10.1371/journal.pone.0185156 (PMC5612684; doi:10.1371/journal.pone.0185156)

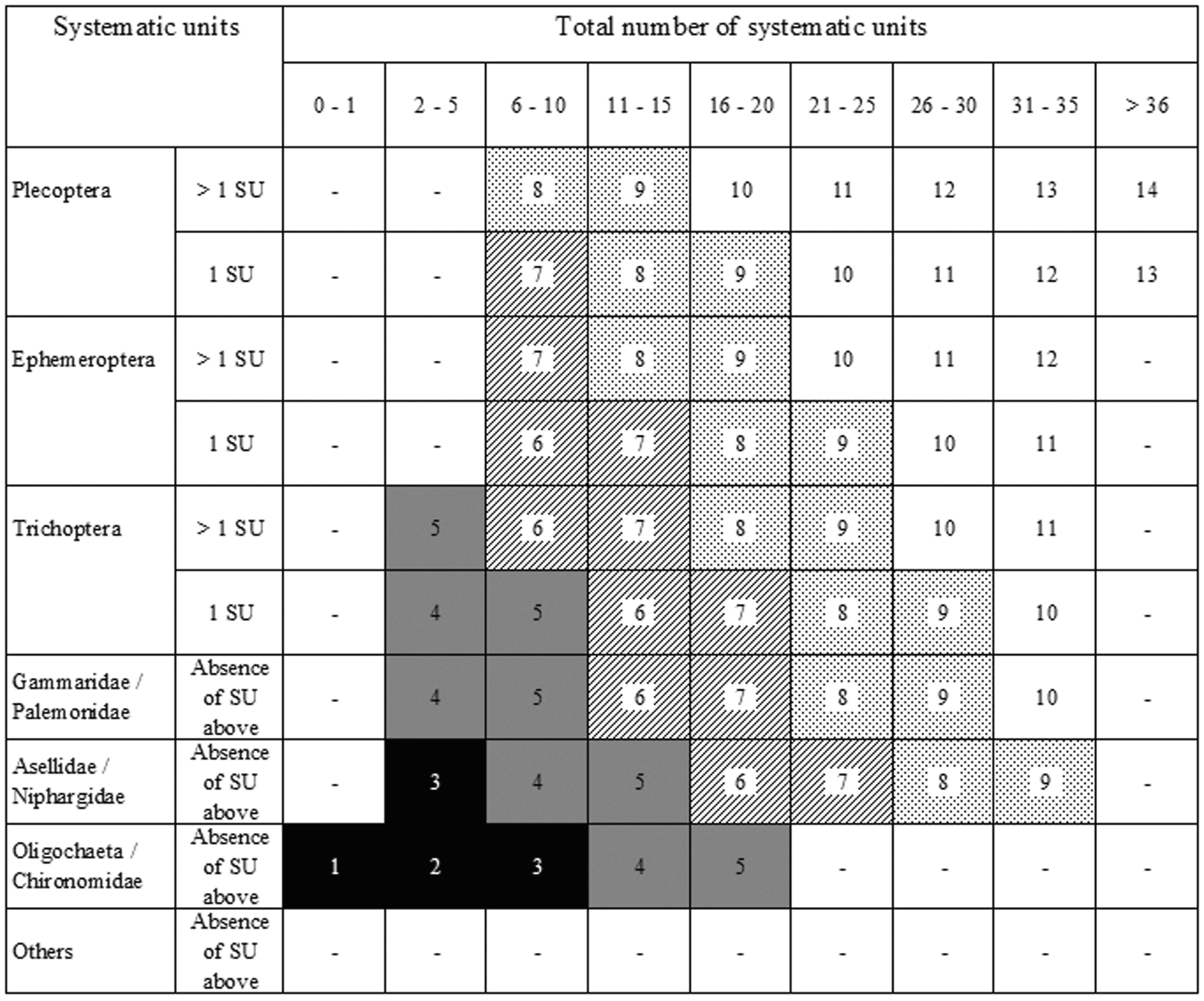

Supplement: S1 Fig — (TIF) [file pone.0185156.s001.tif]
