# Supplementary material for: Integrating running water monitoring tools with the Micro Biological Survey (MBS) method to improve water quality assessment
Source: PLoS One. 2017 Sep 25;12(9):e0185156. doi: 10.1371/journal.pone.0185156 (PMC5612684; doi:10.1371/journal.pone.0185156)
Supplement: S1 Table — Above: bacterial concentrations (expressed as log CFU/ml) obtained with the reference method and corresponding times (expressed in hours) occurred for colour change obtained with the MBS method analyzing identical surface water samples. Below: bacterial concentrations (expressed as log CFU/ml) obtained with the reference method and corresponding bacterial concentrations (expressed as log CFU/ml) obtained with the MBS method analyzing identical surface water samples. Each value is the mean of at least three different analyses. (DOC) [file pone.0185156.s002.doc]

| Linearity | | | | | |
| --- | --- | --- | --- | --- | --- |
| heterotrophic bacteria count 22°C | | heterotrophic bacteria count 37°C | | *E. coli* | |
| time (hrs) | log (CFU/ml) | time (hrs) | log (CFU/ml) | time (hrs) | log (CFU/ml) |
| 13.56 | 4.06 | 6.96 | 3.67 | 8.19 | 3.05 |
| 13.87 | 4.01 | 8.54 | 4.64 | 8.19 | 2.67 |
| 15.82 | 3.97 | 8.24 | 4.29 | 9.21 | 2.67 |
| 17.22 | 4.34 | 9.72 | 4.22 | 9.91 | 2.66 |
| 20.82 | 3.46 | 10.26 | 3.98 | 10.99 | 2.17 |
| 21.03 | 3.55 | 11.00 | 3.09 | 11.80 | 2.30 |
| 21.75 | 3.56 | 13.27 | 3.72 | 12.55 | 2.16 |
| 24.23 | 3.44 | 12.48 | 2.94 | 13.25 | 2.68 |
| 23.81 | 3.18 | 13.03 | 2.56 | 15.19 | 2.65 |
| 26.91 | 3.55 | 12.88 | 2.47 | 13.57 | 2.20 |
| 24.74 | 3.36 | 13.13 | 2.41 | 14.06 | 1.92 |
| 25.15 | 3.29 | 13.13 | 2.25 | 14.49 | 1.98 |
| 25.57 | 3.30 | 14.16 | 2.52 | 15.30 | 1.82 |
| 26.29 | 3.18 | 15.15 | 1.79 | 15.24 | 1.62 |
| 27.27 | 2.99 | - | - | 16.75 | 1.88 |
| 28.09 | 2.92 | - | - | - | - |
| 27.78 | 2.81 | - | - | - | - |
| Accuracy | | | | | |
| heterotrophic bacteria count 22°C | | heterotrophic bacteria count 37°C | | *E. coli* | |
| log (CFU/ml) REF | log (CFU/ml) MBS | log (CFU/ml) REF | log (CFU/ml) MBS | log (CFU/ml) REF | log (CFU/ml) MBS |
| 2.63 | 2.80 | 1.81 | 2.26 | 1.62 | 1.86 |
| 2.86 | 2.90 | 2.32 | 2.76 | 1.88 | 1.58 |
| 2.96 | 2.97 | 2.38 | 2.90 | 1.89 | 1.82 |
| 2.84 | 3.17 | 2.58 | 2.93 | 1.98 | 1.93 |
| 2.95 | 3.18 | 2.45 | 2.76 | 2.11 | 1.88 |
| 3.07 | 3.14 | 2.61 | 2.77 | 1.91 | 2.15 |
| 3.13 | 3.38 | 2.58 | 2.57 | 2.44 | 1.59 |
| 3.38 | 3.52 | 2.41 | 2.45 | 2.77 | 1.60 |
| 3.50 | 3.54 | 2.97 | 2.98 | 2.15 | 2.12 |
| 3.54 | 3.34 | 3.08 | 3.32 | 2.16 | 2.32 |
| 3.52 | 3.23 | 3.72 | 2.76 | 2.33 | 2.14 |
| 3.55 | 3.14 | 4.21 | 3.71 | 2.56 | 2.16 |
| 3.68 | 3.50 | 3.66 | 4.43 | 2.18 | 2.63 |
| 3.88 | 3.46 | 4.21 | 4.08 | 2.41 | 2.62 |
| 4.10 | 4.29 | 4.34 | 4.04 | 2.69 | 2.61 |
| 4.25 | 4.03 | 4.59 | 4.06 | 2.83 | 2.63 |
| 4.22 | 3.92 | - | - | 3.00 | 2.61 |
