# Supplementary material for: Integrating running water monitoring tools with the Micro Biological Survey (MBS) method to improve water quality assessment
Source: PLoS One. 2017 Sep 25;12(9):e0185156. doi: 10.1371/journal.pone.0185156 (PMC5612684; doi:10.1371/journal.pone.0185156)
Supplement: S2 Table — Marks: BOD = biochemical oxygen demand (mg/l); C = conductivity (μS/cm); COD = chemical oxygen demand (mg/l); NH4+ = ammonium (mg/l); NO3- = nitrates (mg/l); P = total phosphorus (mg/l); SAT = saturation (%); T = temperature (°C); VEL = velocity (cm/s). For site marks, see the Fig 1 caption. (DOC) [file pone.0185156.s003.doc]

|  | O_2_ | SAT | pH | T | C | NO_3_^-^ | P | NH_4_^+^ | BOD_5_ | COD | VEL |
| --- | --- | --- | --- | --- | --- | --- | --- | --- | --- | --- | --- |
| ANI1 | 7.05 | 79 | 7.23 | 18.5 | 1260 | 0.81 | 0.15 | 0.20 | 6.1 | 7.12 | 42 |
| ARR1 | 9.22 | 95 | 8.02 | 18.7 | 683 | 7.31 | 1.57 | 0.01 | 8.5 | 28.00 | 23 |
| ARR2 | 9.83 | 94 | 8.15 | 21.0 | 839 | 4.68 | 0.50 | 0.02 | 6.4 | 19.10 | 2 |
| FAR1 | 10.13 | 101 | 8.15 | 14.0 | 527 | 0.90 | 0.12 | 0.02 | 1.8 | 2.20 | 51 |
| FAR2 | 9.03 | 99 | 8.21 | 18.2 | 460 | 0.81 | 0.04 | 0.01 | 1.9 | 0.47 | 25 |
| LIR1 | 8.99 | 94 | 8.02 | 17.1 | 611 | 0.69 | 0.04 | 0.01 | 5.9 | 19.00 | 3 |
| MAR1 | 8.54 | 108 | 8.85 | 25.7 | 532 | 0.06 | 0.10 | 0.02 | 4.2 | 14.90 | 68 |
| MAR2 | 7.33 | 95 | 8.55 | 28.5 | 711 | 4.23 | 0.38 | 0.02 | 4.6 | 11.30 | 5 |
| MIG1 | 6.12 | 69 | 7.12 | 18.3 | 368 | 0.49 | 0.08 | 0.02 | 2.5 | 7.62 | 46 |
| MIG2 | 8.72 | 118 | 8.29 | 31.1 | 625 | 0.10 | 0.07 | 0.02 | 3.1 | 10.30 | 3 |
| SAC1 | 7.45 | 71 | 7.79 | 17.5 | 600 | 9.03 | 0.32 | 0.02 | 9.6 | 31.20 | 3 |
| SAL1 | 11.77 | 144 | 8.38 | 21.8 | 454 | 0.12 | 0.11 | 0.01 | 4.5 | 6.79 | 47 |
| SAL2 | 9.90 | 110 | 8.52 | 18.4 | 397 | 0.33 | 0.05 | 0.02 | 2.8 | 5.48 | 46 |
| TRE1 | 9.06 | 82 | 7.09 | 17.0 | 542 | 3.27 | 0.65 | 0.01 | 3.7 | 8.82 | 83 |
| TRE2 | 8.20 | 80 | 7.10 | 22.0 | 652 | 3.15 | 0.73 | 0.04 | 4.5 | 15.50 | 10 |
| VEL1 | 8.63 | 91 | 8.30 | 13.5 | 456 | 0.34 | 0.09 | 0.08 | 1.8 | 4.39 | 56 |
| VEL2 | 9.99 | 103 | 7.20 | 14.2 | 787 | 0.45 | 0.05 | 0.01 | 2.9 | 5.70 | 84 |
