# Supplementary material for: Integrating running water monitoring tools with the Micro Biological Survey (MBS) method to improve water quality assessment
Source: PLoS One. 2017 Sep 25;12(9):e0185156. doi: 10.1371/journal.pone.0185156 (PMC5612684; doi:10.1371/journal.pone.0185156)
Supplement: S3 Table — Protocols followed national standards and no protected taxa were collected. (DOC) [file pone.0185156.s004.doc]

|  | ANI1 | ARR1 | ARR2 | FAR1 | FAR2 | LIR1 | MAR1 | MAR2 | MIG1 | MIG2 | SAC1 | SAL1 | SAL2 | TRE1 | TRE2 | VEL1 | VEL2 |
| --- | --- | --- | --- | --- | --- | --- | --- | --- | --- | --- | --- | --- | --- | --- | --- | --- | --- |
| *Ancylus* |  |  |  |  |  |  |  |  |  |  |  |  |  |  |  |  |  |
| Anthomyiidae |  |  |  |  |  |  |  |  |  |  |  |  |  |  |  |  |  |
| Asellidae |  |  |  |  |  |  |  |  |  |  |  |  |  |  |  |  |  |
| Athericidae |  |  |  |  |  |  |  |  |  |  |  |  |  |  |  |  |  |
| *Baetis* |  |  |  |  |  |  |  |  |  |  |  |  |  |  |  |  |  |
| Bithynidae |  |  |  |  |  |  |  |  |  |  |  |  |  |  |  |  |  |
| Brachycentridae |  |  |  |  |  |  |  |  |  |  |  |  |  |  |  |  |  |
| *Caenis* |  |  |  |  |  |  |  |  |  |  |  |  |  |  |  |  |  |
| *Calopteryx* |  |  |  |  |  |  |  |  |  |  |  |  |  |  |  |  |  |
| Ceratopogonidae |  |  |  |  |  |  |  |  |  |  |  |  |  |  |  |  |  |
| Chironomidae |  |  |  |  |  |  |  |  |  |  |  |  |  |  |  |  |  |
| *Dina* |  |  |  |  |  |  |  |  |  |  |  |  |  |  |  |  |  |
| *Dinocras* |  |  |  |  |  |  |  |  |  |  |  |  |  |  |  |  |  |
| *Dugesia* |  |  |  |  |  |  |  |  |  |  |  |  |  |  |  |  |  |
| Dytiscidae |  |  |  |  |  |  |  |  |  |  |  |  |  |  |  |  |  |
| *Ecdyonurus* |  |  |  |  |  |  |  |  |  |  |  |  |  |  |  |  |  |
| Elmintidae |  |  |  |  |  |  |  |  |  |  |  |  |  |  |  |  |  |
| *Ephemera* |  |  |  |  |  |  |  |  |  |  |  |  |  |  |  |  |  |
| *Erpobdella* |  |  |  |  |  |  |  |  |  |  |  |  |  |  |  |  |  |
| Gammaridae |  |  |  |  |  |  |  |  |  |  |  |  |  |  |  |  |  |
| Girinidae |  |  |  |  |  |  |  |  |  |  |  |  |  |  |  |  |  |
| *Glossiphonia* |  |  |  |  |  |  |  |  |  |  |  |  |  |  |  |  |  |
| *Habrophlebia* |  |  |  |  |  |  |  |  |  |  |  |  |  |  |  |  |  |
| Haplotaxidae |  |  |  |  |  |  |  |  |  |  |  |  |  |  |  |  |  |
| Hydropsichidae |  |  |  |  |  |  |  |  |  |  |  |  |  |  |  |  |  |
| *Isoperla* |  |  |  |  |  |  |  |  |  |  |  |  |  |  |  |  |  |
| *Lestes* |  |  |  |  |  |  |  |  |  |  |  |  |  |  |  |  |  |
| *Leuctra* |  |  |  |  |  |  |  |  |  |  |  |  |  |  |  |  |  |
| Limnephilidae |  |  |  |  |  |  |  |  |  |  |  |  |  |  |  |  |  |
| Limoniidae |  |  |  |  |  |  |  |  |  |  |  |  |  |  |  |  |  |
| Lumbricidae |  |  |  |  |  |  |  |  |  |  |  |  |  |  |  |  |  |
| Lymnatidae |  |  |  |  |  |  |  |  |  |  |  |  |  |  |  |  |  |
| Naididae |  |  |  |  |  |  |  |  |  |  |  |  |  |  |  |  |  |
| Odontoceridae |  |  |  |  |  |  |  |  |  |  |  |  |  |  |  |  |  |
| Perla |  |  |  |  |  |  |  |  |  |  |  |  |  |  |  |  |  |
| Physidae |  |  |  |  |  |  |  |  |  |  |  |  |  |  |  |  |  |
| *Pisidium* |  |  |  |  |  |  |  |  |  |  |  |  |  |  |  |  |  |
| *Planaria* |  |  |  |  |  |  |  |  |  |  |  |  |  |  |  |  |  |
| *Platycnemis* |  |  |  |  |  |  |  |  |  |  |  |  |  |  |  |  |  |
| Psychodidae |  |  |  |  |  |  |  |  |  |  |  |  |  |  |  |  |  |
| Rhyacophilidae |  |  |  |  |  |  |  |  |  |  |  |  |  |  |  |  |  |
| *Rhytrogena* |  |  |  |  |  |  |  |  |  |  |  |  |  |  |  |  |  |
| *Serratella* |  |  |  |  |  |  |  |  |  |  |  |  |  |  |  |  |  |
| Simulidae |  |  |  |  |  |  |  |  |  |  |  |  |  |  |  |  |  |
| *Sphaerium* |  |  |  |  |  |  |  |  |  |  |  |  |  |  |  |  |  |
| *Theodoxus* |  |  |  |  |  |  |  |  |  |  |  |  |  |  |  |  |  |
| Tipulidae |  |  |  |  |  |  |  |  |  |  |  |  |  |  |  |  |  |
| Tubificidae |  |  |  |  |  |  |  |  |  |  |  |  |  |  |  |  |  |
| Viviparidae |  |  |  |  |  |  |  |  |  |  |  |  |  |  |  |  |  |
| Total number of S.U. | 4 | 7 | 8 | 11 | 12 | 4 | 7 | 3 | 12 | 3 | 10 | 7 | 11 | 15 | 3 | 17 | 10 |
